# Inhibition of HIV-1 subtype C by 2’F-RNA aptamers isolated against enveloped pseudovirus

**DOI:** 10.1186/1742-4690-9-S2-P222

**Published:** 2012-09-13

**Authors:** GG London, MM Madiga, EE Gray, DD Sok, LL Morris, DD Burton, MM Khati

**Affiliations:** 1 CSIR, Biosciences, Pretoria, South Africa; 2 National Institute for Communicable Diseases, Johannesburg, South Africa; 3 The Scripps Research Institute, San Diego, CA, USA; 4 Department of Immunology, The Scripps Research Institute, San Diego, CA, USA; 5 CSIR Biosciences, Pretoria, South Africa

## Background

Human immunodeficiency virus type-I (HIV-1) envelope glycoprotein (Env) mediates the first step of entry and represents an attractive target . However, the genetic diversity of Env among HIV-1 subtypes poses a challenge. Although evidence suggest that entry inhibitors in clinical use have comparable efficacy against HIV-1 subtypes, but a subset of anti-Env are resistant to HIV-1 subtype C which is the most globally prevalent virus. In a view to prevent infection of HIV-1 subtype C, we isolated nucleic acids ligands aptamers against HIV-1 subtype C Env pseudovirion and tested their efficacy.

## Methods

We isolated 109 unique 2`F-RNA aptamer sequences against HIV-1 subtype C Env pseudovirus using systematic evolution of ligands by exponential enrichment (SELEX) process. After 9 selex cycles, we grouped aptamer clones by binding activity to gp120 then selected one of the aptamer which neutralized infection of parental virus with IC50 values <5 nM. We tested efficacy of a selected aptamer against 31-pseudovirus panel from subtype C using single cycle luciferase assay in TZM-bl cells. In addition, we mapped the epitope on gp120 binding by competition.

## Results

When screened on a 31-panel of HIV-1 subtype C pseudoviruses, RNA aptamer CSIR 1.1, neutralized 26 /31 (84%) with mean IC50 of 6.7 ± 8.8 nM. Competition ELISA results revealed CSIR 1.1 bind to the epitope overlapping CD4 and VRC01 on monomeric gp120 but not b12 or b6. In contrast to CD4, CSIR 1.1 did not enhance the binding of 17 b to the co-receptor region on monomeric gp120 showing a different binding mode to the soluble CD4.

## Conclusion

Our data show that we have isolated new RNA aptamers with efficacy against diverse HIV-1 subtype C viruses. The immediate future research would include testing efficacy on primary isolates and precise mapping.

